# Tutor-postgraduate interaction and dropout intention among Chinese medical postgraduates: multiple mediating roles of emotional regulatory self-efficacy and burnout

**DOI:** 10.3389/fpsyg.2025.1580244

**Published:** 2025-10-02

**Authors:** Xue Jiang, Jing Tong, Xiaoning Zhang, Haiyang Li

**Affiliations:** ^1^School of Stomatology, Xuzhou Medical University, Xuzhou, China; ^2^School of Public Health, Xuzhou Medical University, Xuzhou, China; ^3^Shenyang Heping District Disease Prevention and Control Centers, Shenyang, China; ^4^School of Nursing, Hangzhou Normal University, Hangzhou, China; ^5^Zhejiang Philosophy and Social Science Laboratory for Research in Early Development and Childcare, Hangzhou Normal University, Hangzhou, China; ^6^Institute of Medical Humanities, Xuzhou Medical University, Xuzhou, China

**Keywords:** tutor-postgraduate interaction, dropout intention, emotional regulatory self-efficacy, burnout, multiple mediating

## Abstract

**Introduction:**

Tutor-postgraduate interaction plays a significant role in the cultivation of medical postgraduates. However, the relationship between tutor-postgraduate interaction and dropout intention remains poorly understood, and the pathways and mechanisms driving this relationship have yet to be clarified. This study aimed to explore pathways from tutor-postgraduate interaction to dropout intention among Chinese medical postgraduates, and examine the potential mediating roles of burnout and emotional regulatory self-efficacy (ERSE).

**Methods:**

In this cross-sectional study, 1,166 medical postgraduates from a Chinese medical university were recruited between October 1st and December 31st, 2023. Participants completed self-reported questionnaires assessing tutor-postgraduate interaction, burnout, ERSE, dropout intention, and sociodemographic characteristics. Structural equation modeling (SEM) was employed to examine both direct and indirect pathways from tutor-postgraduate interaction to dropout intention via ERSE and burnout.

**Results:**

The prevalence of dropout intention was 19.04% in this study. SEM analysis revealed significant direct (*β* = −0.161, *p* < 0.01) and indirect (*β* = −0.178, *p* < 0.01) associations between tutor-postgraduate interaction and dropout intention. Tutor-postgraduate interaction was indirectly associated with dropout intention via ERSE (*β* = −0.064) and burnout (*β* = −0.055), explaining 18.88 and 16.22% of the total effect, respectively. A sequential mediation pathway via ERSE and burnout (*β* = −0.059) was also identified, accounting for 17.40% of the total effect.

**Conclusion:**

This is the first study to provide empirical evidence for mediating roles of ERSE and burnout in the relationship between tutor-postgraduate interaction and dropout intention among Chinese medical postgraduates. Enhancing ERSE and alleviating burnout represent promising targets for preventive interventions aimed at reducing dropout intention. These findings offer valuable insights for improving tutorship and informing relevant educational policies in Chinese medical postgraduate education.

## 1 Introduction

Dropout has emerged as a complex and global concern in medical education, referring to students who discontinue school studies without completing their degrees (Bernardo et al., 2022; O’Neill et al., 2011; Pedditzi et al., 2022). Dropping out of medical students represents a significant loss of potential contributions to medicine (Haakenstad et al., 2022), imposing substantial financial burdens for both students and institutions, undermining students’ self-confidence and diminishing the quality of research and teaching (O’Neill et al., 2011). These consequences ultimately compromise healthcare services and societal welfare (Abreu Alves et al., 2022).

Given the high stakes of dropout for medical students, institutions, and society (O’Neill et al., 2011), growing studies have instead focused on dropout intention—a preventive lens that advances the understanding of this phenomenon prior to actual dropout behavior emerging (Pedditzi, 2024). Dropout intention refers to students’ thoughts, desires and intentions during their university studies to discontinue degree programs or leave higher education before graduation (Lopez-Angulo et al., 2022; Mashburn, 2000), which has been extensively studied among undergraduates in Western countries. For instance, a survey of 2,222 American medical students has revealed that 25.2% reported dropout intentions, with 11% seriously considering dropping out annually (Dyrbye et al., 2010). In China, a cross-sectional study of 1,383 medical undergraduates has shown that 39.1% developed dropout intentions within the past year (Peng et al., 2023). With the reforms in medical postgraduate education and the implementation of standardized resident training (SRT), as a distinct group facing unique challenges, Chinese medical postgraduates have become indispensable to high-quality healthcare workforce (Peng et al., 2022; Xiao et al., 2021). However, there remains a paucity of information regarding the dropout intention of Chinese medical postgraduates, despite the critical implications of their graduation for healthcare workforce sustainability (Dyrbye et al., 2010).

Dropout intention emerges from a dynamic decision-making process that unfolds during early stages of higher education, shaped by interactive influences of psychological attributes and environmental factors (Stinebrickner and Stinebrickner, 2014; Tinto, 1975). Drawing on Tinto’s theory, dropout intention is primarily driven by perceived “academic integration” and “social integration” within the educational environment (Tinto, 1975), a process where educator’s role requires particular attention (Lopez-Angulo et al., 2022). In the context of Chinese medical postgraduate education, these integrations are profoundly influenced by the tutor responsibility system, where tutors serve as clinical trainers, research supervisors, and professional role models (Xiao et al., 2021). This system assigns tutors pivotal responsibilities throughout the postgraduate cultivation (Su et al., 2023). Tutor support has a stronger effect than family or peer support (Chu et al., 2010), providing more practical and direct assistance (Kim et al., 2018). Interactions with tutors fulfill multiple functions, encompassing academic guidance, career counseling, and emotional support (Le et al., 2021), which are vital for consolidating postgraduates’ career interests and fostering professional identity (Matthew, 2018). High-quality tutor-postgraduate interactions has been linked to academic achievements, professional growth and personal well-beings, thereby strengthening postgraduates’ professional commitment and educational aspirations (Rimm-Kaufman et al., 2015; Su et al., 2023; Trolian and Parker, 2017). Consequently, initial evidence suggests that tutor-postgraduate interaction may play an important role in dropout intention among Chinese medical postgraduates.

Grounded in the Theory of Planned Behavior (TPB), as a direct precursor to actual dropout behavior, dropout intention is influenced by behavioral attitudes, subjective norms, and perceived behavioral control (Lopez-Angulo et al., 2022). Self-efficacy and self-regulation have been identified as significant predictors of dropout intention (Alivernini and Lucidi, 2011; Morelli et al., 2023). Emotional regulatory self-efficacy (ERSE), a domain-specific application of self-efficacy theory, refers to individuals’ subjective self-assessment of their abilities to express positive affect and regulate negative emotions (Alessandri et al., 2015; Bandura et al., 2003; Caprara et al., 2008). This construct highlights the role of self-efficacy in emotional management, serving as a key regulator of personality, behavior and mental health (Mao et al., 2022), which are important for academic persistence. Functioning as a core mechanism in emotional self-regulation, ERSE not only directly influences behavioral outcomes, but also mediates them through cognitive, motivational, and emotional processes (Dongling et al., 2010). Prior research has linked ERSE to psychological distress among medical students (Zhang et al., 2022), and demonstrated its mediating role between post-traumatic stress symptoms and suicide risk in graduate populations (Zeng et al., 2018). Therefore, it is necessary to examine the potential mediating role of ERSE in developing dropout intention among Chinese medical postgraduates considering their unique challenges.

Burnout, a well-established risk factor for dropout intention, is highly prevalent in medical education settings (Jackson-Koku and Grime, 2019; Rosales-Ricardo et al., 2021), and consistently linked to general psychological distress, emotional dysregulations and dropping out (Gorgens-Ekermans and Brand, 2012; Jackson-Koku and Grime, 2019; Maroco et al., 2020). The personal, psychological, and financial consequences of burnout and dropout have been found to be relevant (Abreu Alves et al., 2022). For instance, American medical students with burnout exhibit a 7% increased risk of serious dropout intention in the following year (Dyrbye et al., 2010). Cross-cultural studies have revealed striking regional disparities: the strongest association between burnout and dropout intention was observed among medical students in Western countries (e.g., Portugal and England), while the weakest was reported in Africa (e.g., Mozambique) (Maroco et al., 2020). These disparities may be potentially attributable to cultural value differences of higher education and institutional support systems (Maroco et al., 2020). Notably, a recent Chinese study found that 60% of medical postgraduates reported dropout intention within the past year, with high rates of co-occurring depression, anxiety and burnout (Peng et al., 2022). Given these findings, it is essential to examine the role of burnout in developing dropout intention among Chinese medical postgraduates.

Research has established significant associations between high-quality student-teacher interactions and students’ self-efficacy (Defreitas and Antonio, 2012), emotional regulation (Gavriluță et al., 2022) and graduate aspirations (Hanson et al., 2016), while inadequate relationships and interactions negatively influence these domains (Pedditzi, 2024). Favorable tutor-postgraduate interactions and relationships provide important psychosocial buffers against psychological disorders (e.g., anxiety, burnout) and negative emotions for medical postgraduates (Kim et al., 2018; Yao et al., 2022). Rooted in self-efficacy theory, ERSE not only correlates with psychosomatic disorders (Ewing et al., 2019), but also significantly shapes mental health and adaptive behaviors by mediating emotional management strategies (Mao et al., 2022; Zeng et al., 2023). Meta-analytic findings have identified emotional dissonance and negative emotions as predictors of burnout (Hülsheger and Schewe, 2011), particular among medical students (Gagnon et al., 2016). Building on these theoretical frameworks and empirical findings, ERSE and burnout are hypothesized to mediate the relationship between tutor-postgraduate interaction and dropout intention among Chinese medical postgraduates.

**Figure 1:**
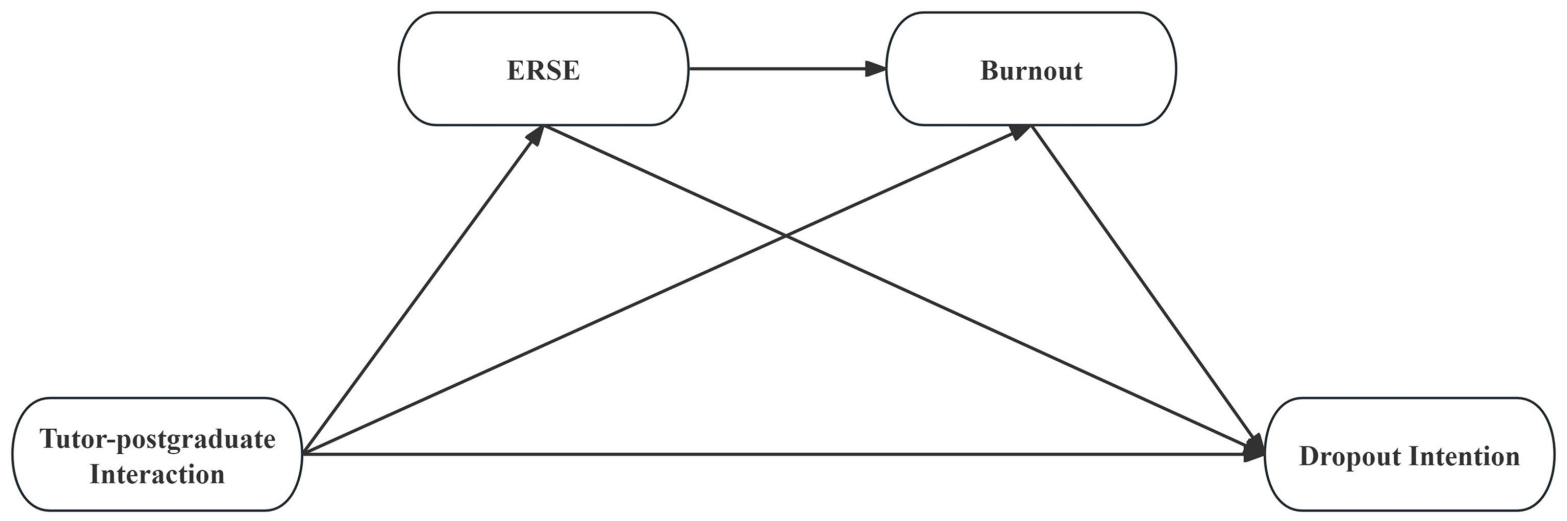
Conceptual model of the relationship between tutor-postgraduate interaction and dropout intention. ERSE, Emotional Regulatory Self-Efficacy.

Despite growing interest in dropout intention, ERSE, burnout, and tutor-postgraduate interactions, significant research gaps persist regarding their associations. Psychosocial resources from tutor-postgraduate interactions—including academic guidance, emotional support, and professional modeling—have drawn scholarly attention (Le et al., 2021), empirical studies examining their protective roles against dropout intention among medical postgraduates remains scarce. The mechanisms and pathways from tutor-postgraduate interactions to dropout intention via ERSE and burnout within the unique context of Chinese medical postgraduates are yet to be fully delineated. Based on the aforementioned theories combined with literature review, this study proposes a conceptual framework (Figure 1) and following hypotheses:

*H1*: Tutor-postgraduate interaction is directly associated with dropout intention among medical postgraduates.

*H2*: Burnout mediates the relationship between tutor-postgraduate interaction and dropout intention.

*H3*: ERSE mediates the relationship between tutor-postgraduate interaction and dropout intention.

*H4*: Tutor-postgraduate interaction influences dropout intention through a chain mediation pathway of ERSE and burnout.

## 2 Methods

### 2.1 Study design and participants

This cross-sectional study utilized convenience sampling to recruit medical postgraduates from Xuzhou Medical University, Jiangsu Province, China, from October 1 to December 31, 2023. A structured questionnaire was administered through the online survey platform Wenjuanxing,^1^ with an estimated completion time of 20–25 min. We did not strictly limit the response time but emphasized authenticity and thoughtfulness to minimize potential biases. Responses submitted in less than 3 min were excluded, yielding a final sample of 1,166 participants. The online platform was programmed to prevent participants from proceeding to the next question without providing an answer. As such, there was no missing data in this study. Prior to participation, all participants received detailed information regarding study’s objectives, procedures, risks, ethics approval, and data confidentiality policies. Informed consent was electronically obtained through an opt-in procedure, with explicit reminders of participants’ rights to withdraw from the study at any time without penalty. The study protocol adhered to the Declaration of Helsinki and was approved by the ethics committee of the Institutional Review Board at Xuzhou Medical University (ID number: XZHMU-2023118).

### 2.2 Measures

The online self-reported questionnaire comprised four sections: tutor-postgraduate interaction, ERSE, burnout, dropout intention, and sociodemographic characteristics. These variables were selected based on established psychometric properties, frequency of use with medical student populations, and feasibility for online administration (Abreu Alves et al., 2022; Dyrbye et al., 2010; Peng et al., 2022).

#### 2.2.1 Sociodemographic characteristics

Sociodemographic characteristics included age, gender, academic year, academic performance and degree type.

#### 2.2.2 Dropout intention

Dropout intention was assessed based on the responses to the question: “Have you ever thought about dropping out of medical school?” and dichotomized into “Yes” or “No.” This item was selected to filter participants who considered the possibility of dropping out of medical schools independently of the level of seriousness of the thoughts, which has been widely used and validated (Abreu Alves et al., 2022).

#### 2.2.3 Tutor-postgraduate interaction

The Tutors-Postgraduates Interaction scale, developed by Harbin Medical University, was adopted in this study. This 14-item instrument employs a 5-point Likert scale (1 “very inconsistent” to 5 “very consistent”) and encompasses two dimensions: Professional Ability Interaction (7 items, e.g., “The tutor is very strict with your scientific research”) and Comprehensive Cultivation Interaction (7 items, e.g., “The tutor often chats with you”) (Wang et al., 2023). The former dimension captures interactions between tutors and postgraduates within the scope of developing scientific research and clinical skills, focusing on tutors’ guidance toward postgraduates’ academic and professional competencies. In contrast, the latter dimension evaluates the interactions extending beyond academic and professional domain, such as ideological guidance, psychosocial support, career counseling, and personal development (Wang et al., 2023). The original validation study reported excellent reliability with Cronbach’s *α* coefficients of 0.938 for Professional Ability Interaction, 0.935 for Comprehensive Cultivation Interaction, and 0.958 for the total scale; the two dimensions explained 74.273% of variance (Wang et al., 2023). In the present study, Cronbach’s α of the total scale was 0.959, with 0.907 for the Professional Ability subscale and 0.963 for the Comprehensive Cultivation subscale. Higher scores indicate higher levels of tutor-postgraduate interactions.

#### 2.2.4 Emotional regulatory self-efficacy (ERSE)

The Chinese version of Caprara’s ERSE scale was applied, which showed good reliability and validity (Bandura et al., 2003; Yujie et al., 2013). This 5-point Likert scale (1 = very inconsistent to 5 = very consistent) consists of 17 items: 11 items assessing perceived self-efficacy in negative affect (NEG; e.g., “I can avoid getting annoyed when others are deliberately picking on me.”) and 6 items assessing perceived self-efficacy in expressing positive affect (POS; e.g., “When something pleasant happens, I will express my pleasure.”). Psychometric validation with 3,257 Chinese graduates demonstrated strong reliability (POS: Cronbach’s α = 0.835; NEG: Cronbach’s α = 0.908) (Zeng et al., 2018). In the present study, Cronbach’s α of the total scale was 0.966, with 0.950 for POS subscale and 0.974 for NEG subscale. Higher scores indicate greater confidence in emotional regulation.

#### 2.2.5 Burnout

Burnout was measured using two single items from the Maslach Burnout Inventory (MBI). This 2-item burnout scale is a validated reliable abbreviated burnout assessment tool that has been widely used in research on psychological distress in medical students (Ernst et al., 2021; Peng et al., 2022; West et al., 2012). Participants were requested to rate their experiences of emotional exhaustion (“How often do you feel burned out from medical learning?”) and depersonalization (“How often do you feel callous toward people since entering medical colleges?”) on a 5-point Likert scale (1 = Never, 5 = Daily). Notably, West et al., demonstrated that single-item test of emotional exhaustion and depersonalization triggers deeper evaluations of distress for individuals while maintaining diagnostic sensitivity at the group level (West et al., 2012). Participants who reported emotional exhaustion or depersonalization at least weekly were classified as experiencing burnout.

### 2.3 Statistical analysis

Data analyses were conducted using SPSS 27.0 for descriptive statistics (e.g., prevalence of dropout intention) and bivariate Spearman’s correlations between tutor-postgraduate interaction, ERSE, burnout and dropout intention. Mplus 7.4 was employed to perform structural equation modelling (SEM) analysis (Muthén and Muthén, 2012). As an advanced approach to estimate relationships among latent variables, SEM has the potential to incorporate multiple variables and disentangle complex pathways simultaneously (Shah and Goldstein, 2006). Based on the conceptual framework (Figure 1), the SEM model was developed to examine hypothesized pathways from tutor-postgraduate interaction and dropout intention. First, a measurement model was specified to assess factor loadings and interrelationships among study variables. Tutor-postgraduate interaction was modeled as a latent variable with two observed indicators: professional ability interaction and comprehensive cultivation interaction. ERSE was also modeled as a latent variable, measured by two subscales (POS and NEG), while burnout was represented by two directly observed single-item measures (emotional exhaustion and depersonalization). Then, the structural model was tested to evaluate ERSE and burnout as mediators in the pathways from tutor-postgraduate interaction to dropout intention. To estimate mediation effects, a bootstrapping procedure with 2,000 resamples was employed to derive bias-corrected 95% confidence intervals (CIs) for indirect effects (Erceghurn and Mirosevich, 2008; Preacher and Hayes, 2008). The total effects (c) were calculated as the sum of direct (c’) and indirect effects (ab), mathematically expressed as (Vanderweele, 2015): c = c’ + ab. Model fit was evaluated using following indices: the root mean square error of approximation (RMSEA), standardized root mean square residual (SRMR), Tucker-Lewis index (TLI), and comparative fit index (CFI), with good model fit defined as: CFI > 0.90, TLI > 0.90, RMSEA < 0.06, and SRMR < 0.06 (Browne and Cudeck, 1992). For all statistical analyses, *α* = 0.05 was applied.

## 3 Results

### 3.1 Descriptive statistics and correlation analyses

As presented in Table 1, the study sample comprised 1,166 medical postgraduate students, of whom 222 (19.04%) reported dropout intention. The sample predominantly included female participants (66.81%), and 74.61% of postgraduates were aged over 25. Degree type was approximately evenly distributed, with 607 (52.06%) participants pursuing professional degrees. Significant differences in dropout intention were observed across academic year (*p* < 0.01) and degree type (*p* < 0.01). Specifically, first-grade postgraduates showed lower dropout intention, while third-grade postgraduates reported higher intention (*p* < 0.01). Postgraduates pursuing academic degrees were more likely to report dropout intention than those pursing professional degrees (*p* < 0.01). There were no significant differences were determined in gender, age group and academic performance between participants with and without dropout intentions (*p* > 0.05).

**Table 1:** Socioeconomic characteristic and dropout intention distribution (*N* = 1,166).

Variables	Dropout intention		χ^2^ value	*p*-value
	Yes	No		
	222 (19.0%)	944 (81.0%)		
Gender				
Male	64 (28.8%)	323 (34.2%)	2.352	0.125
Female	158 (71.2%)	621 (65.8%)		
Age group				
≤25	163 (73.4%)	707 (74.9%)	2.656	0.265
26–30	46 (20.7%)	204 (21.6%)		
≥30	13 (5.9%)	33 (3.5%)		
Academic year				
First grade master	89 (40.1%)	552 (58.5%)	25.217	<0.001
Second grade master	79 (35.6%)	247 (26.2%)		
Third grade master	54 (24.3%)	145 (15.4%)		
Academic performance				
The first third	82 (36.9%)	337 (35.7%)	3.28	0.194
The middle third	100 (45.0%)	385 (40.8%)		
The last third	40 (18.0%)	222 (23.5%)		
Degree type				
Academic degree	129 (58.1%)	430 (45.6%)	11.356	<0.001
Professional degree	93 (41.9%)	514 (54.4%)		

Table 2 showed bivariate correlations between tutor-postgraduate interaction, ERSE, burnout, and dropout intention. Tutor-postgraduate interaction was negatively correlated with burnout (r = −0.288, *p* < 0.01) and dropout intention (r = −0.235, *p* < 0.01). It also showed a positive correlation with ERSE (r = 0.472, *p* < 0.01), particularly with ERSE in regulating negative affect (ERSE_NEG; r = 0.461, *p* < 0.01).

**Table 2:** Correlations between tutor-postgraduate interaction, ERSE, burnout, and dropout intention (*N* = 1,166).

Variables	1	2	3	4	5	6	7	8
1. Tutor-postgraduate interaction	1							
2. Professional ability interaction	0.927**	1						
3. Comprehensive cultivation interaction	0.954**	0.791**	1					
4. ERSE	0.472**	0.415**	0.479**	1				
5. ERSE_POS	0.400**	0.371**	0.392**	0.788**	1			
6. ERSE_NEG	0.461**	0.393**	0.477**	0.949**	0.596**	1		
7. Burnout	−0.288**	−0.246**	−0.289**	−0.421**	−0.337**	−0.403**	1	
8. Dropout intention	−0.235**	−0.179**	−0.255**	−0.233**	−0.095**	−0.266**	0.304**	1

ERSE, Emotional Regulatory Self-Efficacy; POS, perceived self-efficacy in expressing positive affect; NEG, perceived self-efficacy in negative affect. **p < 0.01.

ERSE was negatively associated with burnout (r = −0.421, *p* < 0.01) and dropout intention (r = −0.233, *p* < 0.01), with ERSE_NEG showing a stronger relationship with dropout intention (r = −0.266, *p* < 0.01). Burnout was positively correlated with dropout intention (r = 0.304, *p* < 0.01). These above results indicated significant associations between independent variable (dropout intention), dependent variables (tutor-postgraduate interaction), and mediating variables (ERSE and burnout), preliminarily validating the conceptual model.

### 3.2 SEM results

As shown in Table 3, CFA results confirmed the psychometric properties of the key constructs. All standardized factor loadings of latent constructs (tutor-postgraduate interaction, ERSE and burnout) exceeded 0.60, indicating strong construct validity.

**Table 3:** Standardized loadings based on confirmatory factor analysis for tutor-postgraduate interaction, ERSE and burnout.

Factors		Factor loading
Tutor-postgraduate interaction	Professional ability interaction	0.897
	Comprehensive cultivation interaction	0.997
ERSE	ERSE_POS	0.643
	ERSE_NEG	0.901
Burnout	Emotional exhaustion	0.860
	Depersonalization	0.875

ERSE, Emotional Regulatory Self-Efficacy; POS, perceived self-efficacy in expressing positive affect; NEG, perceived self-efficacy in negative affect.

As presented in Table 4, the measurement model and structural model both showed acceptable fit to the data. The structural model demonstrated satisfactory model fit: RMSEA = 0.039, SRMR = 0.031, CFI = 0.902, and TLI = 0.910; the measurement model also met criteria for good fit: RMSEA = 0.051, SRMR = 0.036, TLI = 0.935 and CFI = 0.940.

**Table 4:** Model fit indices of the hypothesized models (*N* = 1,166).

Models	Fit indices			
	RMSEA	SRMR	TLI	CFI
Measurement model	0.051	0.036	0.935	0.940
Structural model	0.039	0.032	0.910	0.902

RMSEA, root mean square error of approximation; SRMR, standardized root mean square residual; TLI, Tucker-Lewis index; CFI, comparative fit index.

**Figure 2:**
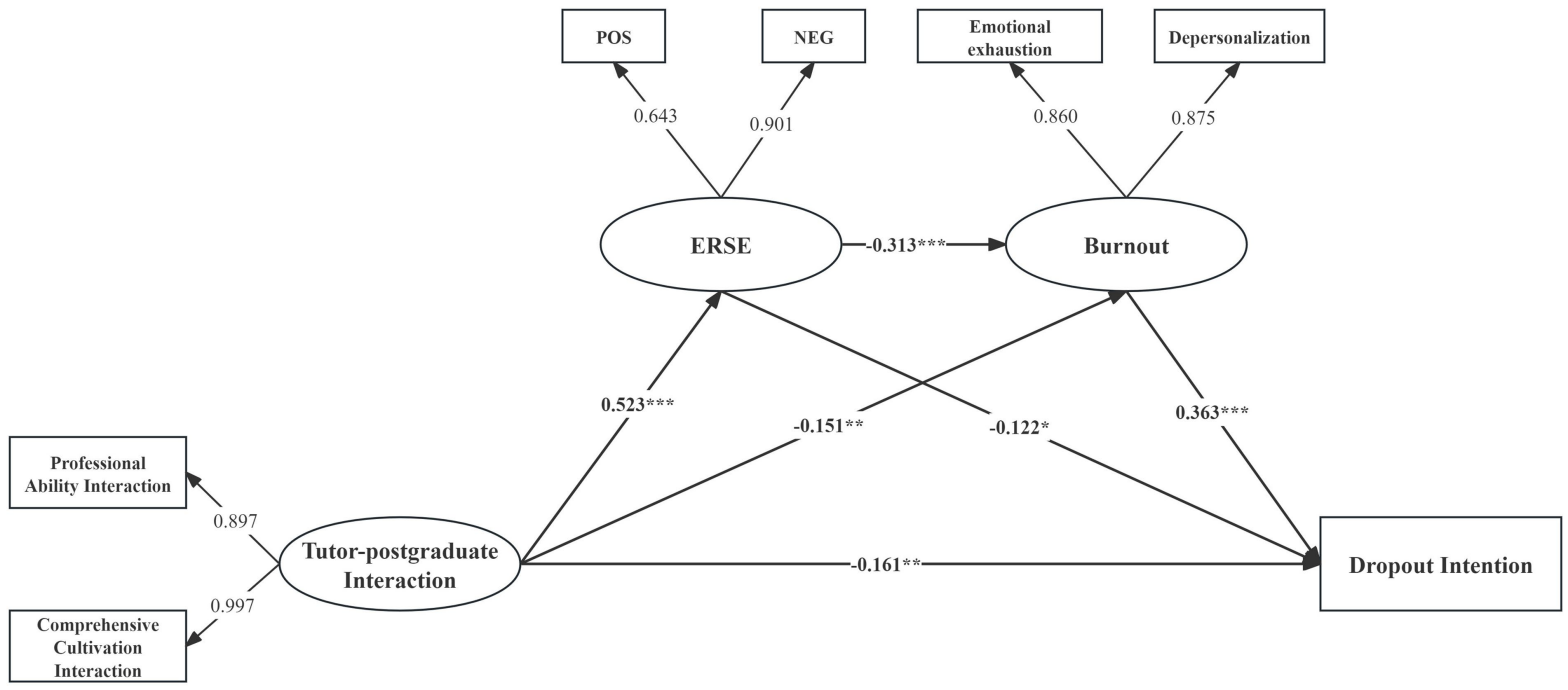
Standardized estimates of path effects from tutor-postgraduate interaction to dropout intention. ERSE, Emotional Regulatory Self-Efficacy. *p < 0.05, **p < 0.01, ***p < 0.001.

Table 5 summarized the standardized direct, indirect and total path estimates from tutor-postgraduate interaction to dropout intention for the structural model. Figure 2 visualized the validated conceptual structural model with solid arrows representing significant paths (*p* < 0.05), illustrating direct and indirect pathways from tutor-postgraduate interaction to dropout intention and mediating roles of ERSE and burnout.

**Table 5:** Total, direct, and indirect estimates from tutor-postgraduate interaction to dropout intention (*N* = 1,166).

Effects	Paths	*β*	S.E.	*P*	BC 95% CI
Direct	Tutor-postgraduate Interaction → ERSE	0.523	0.038	<0.001	[0.447, 0.598]
	Tutor-postgraduate Interaction → Burnout	−0.151	0.047	0.001	[−0.239, −0.053]
	Tutor-postgraduate Interaction → Dropout Intention	−0.161	0.048	0.001	[−0.256, −0.067]
	ERSE → Burnout	−0.313	0.053	<0.001	[−0.422, −0.214]
	ERSE → Dropout Intention	−0.122	0.056	0.030	[−0.231, −0.011]
	Burnout → Dropout Intention	0.363	0.041	<0.001	[0.282, 0.445]
Indirect	Tutor-postgraduate Interaction → ERSE → Dropout Intention	−0.064	0.030	0.034	[−0.125, −0.005]
	Tutor-postgraduate Interaction → Burnout → Dropout Intention	−0.055	0.01	0.002	[−0.089, −0.019]
	Tutor-postgraduate Interaction → ERSE → Burnout → Dropout Intention	−0.059	0.016	<0.001	[−0.095, −0.034]
Total	Tutor-postgraduate Interaction → Dropout Intention	−0.339	0.037	<0.001	[−0.412, −0.267]

ERSE, Emotional Regulatory Self-Efficacy.

The SEM results demonstrated significant direct (*β* = − 0.161, *p* < 0.01) and indirect (*β* = −0.178, *p* < 0.01) associations between tutor-postgraduate interaction and dropout intention, with indirect pathways accounting for 52.51% of the total effects (*β* = −0.339, *p* < 0.01). ERSE was negatively associated with burnout (*β* = −0.313, *p* < 0.01) and dropout intention (*β* = −0.122, *p* < 0.05), while burnout positively associated with dropout intention (*β* = 0.363, *p* < 0.01). Specifically, the indirect pathway through ERSE was estimated at −0.064, accounting for 35.96% of the total indirect effect, while the indirect pathway through burnout was estimated at −0.055, accounting for 30.90% of the total indirect effect. The sequential mediating effect of ERSE and burnout was significant (*β* = −0.059, *p* < 0.01), accounting for 33.15% of the indirect effect. The total effect of tutor-postgraduate interaction on dropout intention was significant and negative (*β* = −0.339, *p* < 0.01), indicating that more favorable tutor-postgraduate interaction was associated with lower dropout intention, partially through the mediating roles of ERSE and burnout.

## 4 Discussion

To the best of our knowledge, this is the first study to examine the pathways and mechanisms linking tutor-postgraduate interaction to dropout intention within a Chinese medical postgraduate sample. Employing the SEM approach, this study identified one direct pathway from tutor-postgraduate interaction to dropout intention and three indirect pathways: single mediating pathway via ERSE, single mediating pathway via burnout, and sequential mediation pathway via ERSE and burnout, among which the ERSE-mediated pathway showing the strongest mediating effect. These findings significantly advanced the theoretical understanding of direct and indirect relationships between tutor-postgraduate interaction, ERSE, burnout and dropout intention. The findings offered evidence-based guidance for policy-making and educational practice, advocating for targeted interventions aimed at improving tutor-postgraduate interactions, promoting ERSE development, and implementing effective burnout prevention strategies, to mitigate dropout intention among Chinese medical postgraduate population.

### 4.1 Direct pathway from tutor-postgraduate interaction to dropout intention

This study confirmed Hypothesis 1, revealing a direct association between inadequate tutor-postgraduate interaction and a higher risk of dropout intention. This finding aligned with previous research demonstrating that strained tutor-student relationships and interactions were significantly linked to dropping out among graduate students (Willis and Carmichael, 2011). Through the course of daily interactions, the relationship between the tutor and postgraduate is well established and developed (Yu et al., 2017). Empirical evidence from Italy further corroborated that dissatisfaction of students with their teacher relationships elevated the risk of dropout intentions (Pedditzi, 2024). Conversely, favorable tutor-postgraduate interactions, characterized by effective teaching, supportive communication and academic guidance (Wang et al., 2023), equip postgraduates with essential competencies like problem-solving skills and emotional resilience. These capacities enable them to navigate training challenges, thereby reducing dropout intentions. Research showed that such high-quality interactions increased the likelihood of persisting advanced degrees (Trolian and Parker, 2017). Specifically, interactions centered on professional development provide postgraduates with insightful and actionable feedback to academic progress, career readiness and professional growth in medicine (Su et al., 2022). Simultaneously, interactions emphasizing comprehensive cultivation, such as ideological mentoring, emotional support, and competency development (Wang et al., 2023), deliver psychological assistance through counselling, acceptance, affirmation, and role-modeling (Stamm and Buddeberg-Fischer, 2011). These dual dimensions of tutor-postgraduate interaction serve as external assistance directly alleviating dropout intention.

The direct pathway from tutor-postgraduate interaction to dropout intention also reinforced leader-member exchange theory, which postulates that high-quality interactions confer postgraduates “insiders” status, affording preferential access to tailed academic guidance, career counseling, and professional networks, which better equip them with enhanced capacities to navigate training challenges, directly mitigating dropout intention (Noy and Ray, 2012; Wang et al., 2022). This finding also aligned with the social support model, which conceptualizes tutors as “weak ties” of postgraduates, bridging them to broader connections, knowledge and sources (Granovetter, 1973; Le et al., 2021). Such weak ties provide postgraduates more informational support, strengthening their persistence in pursuing degrees (Newman et al., 2020; Raposa et al., 2018), thereby reducing dropout intentions.

### 4.2 Mediating roles of ERSE and burnout in the relationship between tutor-postgraduate interaction and dropout intention

Confirming Hypothesis 2, this study found that burnout mediated the association between tutor-postgraduate interaction and dropout intention, which indicated that suboptimal interactions increased the risk of burnout (emotional exhaustion and depersonalization), thereby inducing dropout intention. This finding aligned with multi-institutional research showing that burnout preceded dropout intention (Dyrbye et al., 2010), with similar results observed in Portuguese medical students (Abreu Alves et al., 2022), nurses and business students (Emerson et al., 2023; Leiter and Maslach, 2009). Grounded in social support theory, suboptimal tutor-postgraduate interactions limit access to essential professional support (Noy and Ray, 2012), and compromise personalized guidance and emotional support (Wang et al., 2022; Yu et al., 2017). From a Conservation of Resources (CoR) perspective, such constraints deplete psychological resources of postgraduates, exacerbating emotional exhaustion and depersonalization (Abreu Alves et al., 2022; Yao et al., 2022), which, in turn, erode academic motivation (Dyrbye et al., 2010), triggering dropout intentions. Burnout is one of the major manifestations of psychological disorders that drive professionals turnover in health care, an event analogous to dropping out of students (Dyrbye et al., 2010; Peng et al., 2022), such parallels further highlight the need for interventions targeting both tutor-postgraduate interaction quality and burnout mitigation to reduce dropout intention.

Hypotheses 3 and 4 were validated, establishing ERSE as a significant mediator in the relationship between tutor-postgraduate interaction and dropout intention among Chinese medical postgraduates. Specifically, poor tutor-postgraduate interaction was associated with lower ERSE, subsequently elevating both risk of burnout and dropout intention. Guidance, expectations, and social support provided by tutors constitutes the cornerstone of postgraduates’ self-efficacy development (Wang et al., 2022); positive relationships and interactions buffers emotion dysregulation (Wang et al., 2020). These processes synergistically promoted ERSE of medical postgraduates. Rooted in CoR theory, high-quality tutor-postgraduate interactions serves as an important relational resource that promote internal psychological support maintenance and foster resources accumulation through the “gain spiral effect,” thereby facilitate emotional regulation capacities (Hobfoll et al., 2018; Yao et al., 2022). Consequently, postgraduates with higher level of ERSE exhibit greater confidence in regulating and managing their emotions (e.g., timely adjustment and rapid adaptation to pressures) (Sui et al., 2021; Zhang et al., 2022), whereas low-level ERSE triggers heightened burnout (Yuan et al., 2018). When exposed to significant unpleasant stimuli (e.g., unsatisfied tutor-postgraduate interactions), postgraduates with low ERSE struggle to regulate negative emotions and suffer greater psychological distress, leading to negative thoughts and burnout (Liu et al., 2020; Zeng et al., 2018), and subsequent dropout intentions (Liu et al., 2020; Sui et al., 2021).

Besides, this study confirmed that ERSE was negatively associated with burnout, consistent with previous evidence across different populations (Jackson-Koku and Grime, 2019; Yuan et al., 2018), further establishing protective role of ERSE. Overall, in this study, heightened ERSE attenuated the detrimental effects of inadequate tutor-postgraduate interaction on both burnout and dropout intention, suggesting ERSE act as a protective buffer against burnout and dropout intention (Liu et al., 2020). These findings highlighted ERSE as a key psychological asset for sustaining academic engagement and reducing dropout intentions among Chinese medical postgraduates.

### 4.3 Implications for practice

The findings hold significant implications for stakeholders in medical postgraduate education, focused on relational resource enhancement and psychological resilience cultivation. Institutions should prioritize tutor training programs that integrate competency-based training for tutors, psychologically attuned communication (e.g., active listening, constructive feedback) to foster trusting relationships (Baker and Griffin, 2010; Trolian and Parker, 2017). Structured initiatives to prompt positive and sustainable tutor-postgraduate interactions are recommended (Su et al., 2022). For example, pilot programs like “Clinical-Research Integration Forums” can create structured platforms for tutors to deliver tailored guidance (e.g., research methodology, clinical decision-making) while fostering postgraduates’ capacity to articulate needs, thereby addressing both professional and emotional support demands. These interventions strengthen postgraduates’ motivation to pursue advanced degrees (Huang et al., 2021), thereby mitigating dropout intention.

This study also identified ERSE and burnout as promising interventional targets for their mediating roles in the pathways from tutor-postgraduate interaction to dropout intention. Integrating emotion regulation workshops (e.g., mindfulness, stress reappraisal) into curricula builds psychological resilience (Yao et al., 2022). Institutions are advised to cultivate supportive environments where routinely incorporate emotional check-ins into mentorship, aligning with resource conservation theory’s “gain spiral” mechanism: supportive interactions enhance ERSE, which in turn protects against burnout. This dual focus on academic and emotional support help medical postgraduates navigate relentless pressures inevitably related to medical practice, thereby relieve the burnout and prevent dropout intention.

The findings further highlighted the critical need to address burnout among medical postgraduate students, as burnout emerges at early stages in professional careers and eventually increases the attrition rates of medical personnel (Abreu Alves et al., 2022). A tiered intervention framework is recommended: universal stress-management modules for primary prevention, targeted counseling for postgraduates with emerging burnout systems, and clinical referrals for high-risk cases. Such stepped-care approach aims to disrupt the burnout-dropout cycle by providing tailored support, preventing the progression from dissatisfactory tutor-postgraduate interactions to irreversible dropout.

### 4.4 Limitations and future directions of research

Several limitations need to be acknowledged. First, the cross-sectional design precluded definitive causal inferences, including potential reverse causality (e.g., high dropout intention or low ERSE leading to reduced tutor-postgraduate interaction) or bidirectional effects between key variables. Longitudinal or experimental studies are needed to clarify the temporal sequence and causal relationships between study variables. Second, the single-institution sample limited generalizability, as the findings may not extend to diverse regional contexts, university types (e.g., comprehensive vs. specialized medical institutions), or doctoral student populations. Future research should expand sampling to include broader demographics and academic levels. Third, burnout was measured using two single items in this study, which may limit the findings and their comparability with existing literature (Ernst et al., 2021; West et al., 2012). Nevertheless, the 2-item scale was developed specifically to measure exhaustion and depersonalization in medical professionals, which has been well validated among medical students and showed satisfactory construct validity (Ernst et al., 2021; Peng et al., 2022). Due to limitations in cross-sectional design and simplification of measurement tools, further validation of the mechanism is needed in the future through longitudinal studies, multi center samples, and more comprehensive measurement tools. Forth, reliance on self-reported measures (dropout intention, tutor-postgraduate interaction, ERSE and burnout) is susceptible to social desirability bias and subjective perception biases, although self-reports may be the most valid assessment method for constructs like burnout, considering individual provides the most accurate account of their own internal experiences (Maroco et al., 2020). This study also employed anonymous responses and well-validated cross-cultural scales (e.g., ERSE scale) to mitigate these potential issues. Future research should incorporate multi-source data to validate these findings. Another key limitation is the omission of potential confounding variables – including demographic (e.g., age, gender), academic (e.g., majors, degree type, performance), and psychological factors (e.g., personality traits, social support, economic pressure and personal health status) - that may independently influence both ERSE and burnout. These unconsidered variables could confound the observed associations or act as mediators/moderators within examined pathways linking tutor-postgraduate interaction to dropout intention, thereby limiting the results. While focusing on clarifying mediation pathways between tutor-postgraduate interaction and dropout intention justified a more parsimonious model, future research should incorporate these variables into advanced multivariate models to further refine the conceptual framework and strengthen the validation of the findings.

### 4.5 Strengths

Despite these limitations, this study has some strengths. To the best of our knowledge, this is the first empirical study to elucidate the pathways linking tutor-postgraduate interaction to dropout intention in Chinese medical postgraduate education, a context with unique tutorship dynamics underexplored in global research. By focusing on this specific population, this study provides contextualized insights into how tutor-postgraduate interactions and relationships influence dropout intentions in collectivist cultures. Methodologically, applying the SEM approach enabled nuanced analyses of direct and indirect pathways from tutor-postgraduate interaction to dropout intention, identifying ERSE and burnout as critical mediators. This approach overcomes traditional regression limitations by estimating direct and indirect effects via multiple pathways and identifying mediating factors, thereby informing evidence-based interventions (Zhang et al., 2023).

## 5 Conclusion

This study provides novel theoretical and practical insights by elucidating dual mediation mechanisms of ERSE and burnout in Chinese medical postgraduates via SEM, identifying ERSE as a protective buffer and burnout as a modifiable risk factor in the pathways linking tutor-postgraduate interaction to dropout intention. These findings advance existing literature by demonstrating how tutor-postgraduate interaction influences dropout intention through both direct support and indirect mechanisms of ERSE enhancement or burnout mitigation. Practically, a three-tiered intervention model is proposed: integrating ERSE assessments into academic advising systems to identify students at risk of emotional dysregulation; implementing burnout screening and evidence-based workshops on stress management during high-stakes periods (e.g., thesis defense, clinical rotations); training tutors to embed emotional regulation guidance into supervisory practices to foster high-quality tutor-postgraduate relationships that enhance psychological resources for postgraduates. These recommendations address the specific need for dropout prevention in Chinese medical postgraduate education, while also contributing to the growing body of research on the efficacy of tutor-postgraduate interactions, providing clear directions for future research and policy development.

## Data Availability

The raw data supporting the conclusions of this article will be made available by the authors, without undue reservation.

## Footnotes

^1^ www.wjx.cn

## References

[ref1] Abreu Alves S. Sinval J. Lucas Neto L. Maroco J. Goncalves Ferreira A. Oliveira P. (2022). Burnout and dropout intention in medical students: the protective role of academic engagement. BMC Med. Educ. 22:83. doi: 10.1186/s12909-021-03094-9, PMID: 35130892 PMC8821797

[ref2] Alessandri G. Vecchione M. Caprara G. V. (2015). Assessment of regulatory emotional self-efficacy beliefs: a review of the status of the art and some suggestions to move the field forward. J. Psychoeduc. Assess. 33, 24–32. doi: 10.1177/0734282914550382

[ref3] Alivernini F. Lucidi F. (2011). Relationship between social context, self-efficacy, motivation, academic achievement, and intention to drop out of high school: a longitudinal study. J. Educ. Res. 104, 241–252. doi: 10.1080/00220671003728062

[ref4] Baker V. L. Griffin K. A. (2010). Beyond mentoring and advising: toward understanding the role of faculty “developers” in student success. About Campus 14, 2–8. doi: 10.1002/abc.20002

[ref5] Bandura A. Fau C. G. Barbaranelli C. Barbaranelli C. Fau-Gerbino M. Gerbino M.. (2003). Role of affective self-regulatory efficacy in diverse spheres of psychosocial functioning. Child Dev. 74, 769–782. doi: 10.1111/1467-8624.00567 12795389

[ref6] Bernardo A. B. Galve-González C. Núez J. C. Almeida L. S. (2022). A path model of university dropout predictors: the role of satisfaction, the use of self-regulation learning strategies and students***’*** engagement. Sustainability 14, –1057. doi: 10.3390/su14031057

[ref7] Browne M. W. Cudeck R. (1992). Alternative ways of assessing model fit. Sociol. Methods Res. 21, 230–258. doi: 10.1177/004912419021002005

[ref8] Caprara G. V. Di Giunta L. Eisenberg N. Gerbino M. Pastorelli C. Tramontano C. (2008). Assessing regulatory emotional self-efficacy in three countries. Psychol. Assess. 20, 227–237. doi: 10.1037/1040-3590.20.3.227, PMID: 18778159 PMC2713723

[ref9] Chu P. S. Saucier D. A. Hafner E. (2010). Meta-analysis of the relationships between social support and well-being in children and adolescents. J. Soc. Clin. Psychol. 29, 624–645. doi: 10.1521/jscp.2010.29.6.624

[ref10] Defreitas S. C. Antonio A. (2012). The influence of involvement with faculty and mentoring on the self-efficacy and academic achievement of African American and Latino college students. J. Scholarship Teach. Learn. 12:17242. Available at: https://files.eric.ed.gov/fulltext/EJ992123.pdf

[ref11] Dongling T. Yan D. Guoliang Y. U. Shufeng W. (2010). The regulatory emotional self-efficacy: a new research topic. Adv. Psychol. Sci. 18:598. Available at: https://journal.psych.ac.cn/adps/EN/abstract/abstract552.shtml

[ref12] Dyrbye L. Power D. Durning S. Moutier C. Massie F. Harper W.. (2010). Burnout and serious thoughts of dropping out of medical school: a multi-institutional study. Acad. Med. 85, 94–102. doi: 10.1097/ACM.0b013e3181c46aad 20042833

[ref13] Emerson D. J. Hair J. F. Jr. Smith K. J. (2023). Psychological distress, burnout, and business student turnover: the role of resilience as a coping mechanism. Res. High. Educ. 64, 228–259. doi: 10.1007/s11162-022-09704-9, PMID: 35789581 PMC9243806

[ref14] Erceghurn D. M. Mirosevich V. M. (2008). Modern robust statistical methods: an easy way to maximize the accuracy and power of your research. Am. Psychol. 63, 591–601. doi: 10.1037/0003-066X.63.7.591 18855490

[ref15] Ernst J. Jordan K. D. Weilenmann S. Sazpinar O. Gehrke S. Paolercio F.. (2021). Burnout, depression and anxiety among Swiss medical students - a network analysis. J. Psychiatr. Res. 143, 196–201. doi: 10.1016/j.jpsychires.2021.09.017, PMID: 34500349

[ref16] Ewing L. Hamza C. A. Willoughby T. (2019). Stressful experiences, emotion dysregulation, and nonsuicidal self-injury among university students. J. Youth Adolesc. 48, 1379–1389. doi: 10.1007/s10964-019-01025-y, PMID: 31025157

[ref17] Gagnon M.-C. J. Natalie D. B. Young B. W. (2016). Self-regulation capacity is linked to wellbeing and burnout in physicians and medical students: implications for nurturing self-help skills. Int. J. Wellbeing 6, 101–116. doi: 10.5502/ijw.v6i1.425

[ref18] Gavriluță C. Dalban C. M. Ioan B. G. (2022). Educational, emotional, and social impact of the emergency state of COVID-19 on Romanian university students. Int. J. Environ. Res. Public Health 19:3990. doi: 10.3390/ijerph19073990, PMID: 35409670 PMC8997823

[ref19] Gorgens-Ekermans G. Brand T. (2012). Emotional intelligence as a moderator in the stress-burnout relationship: a questionnaire study on nurses. J. Clin. Nurs. 21, 2275–2285. doi: 10.1111/j.1365-2702.2012.04171.x, PMID: 22788561

[ref20] Granovetter M. S. (1973). The strength of weak ties. Am. J. Sociol. 78, 1360–1380. doi: 10.1086/225469

[ref21] Haakenstad A. Irvine C. M. S. Knight M. Bintz C. Aravkin A. Y. Zheng P.. (2022). Measuring the availability of human resources for health and its relationship to universal health coverage for 204 countries and territories from 1990 to 2019: a systematic analysis for the global burden of disease study 2019. Lancet 399, 2129–2154. doi: 10.1016/S0140-6736(22)00532-3, PMID: 35617980 PMC9168805

[ref22] Hanson J. M. Paulsen M. B. Pascarella E. T. (2016). Understanding graduate school aspirations: the effect of good teaching practices. High. Educ. 71, 735–752. doi: 10.1007/s10734-015-9934-2

[ref23] Hobfoll S. E. Halbesleben J. Neveu J. P. Westman M. (2018). Conservation of resources in the organizational context: the reality of resources and their consequences. Annu. Rev. Organ. Psychol. Organ. Behav. 5, 103–128. doi: 10.1146/ANNUREV-ORGPSYCH-032117-104640

[ref24] Huang Z. Zhang L. Wang J. Xu L. Liu Z. Wang T.. (2021). Social support and subjective well-being among postgraduate medical students: the mediating role of anxiety and the moderating role of alcohol and tobacco use. Heliyon 7:e08621. doi: 10.1016/j.heliyon.2021.e08621, PMID: 34988318 PMC8695259

[ref25] Hülsheger U. R. Schewe A. F. (2011). On the costs and benefits of emotional labor: a meta-analysis of three decades of research. J. Occup. Health Psychol. 16, 361–389. doi: 10.1037/a0022876, PMID: 21728441

[ref26] Jackson-Koku G. Grime P. (2019). Emotion regulation and burnout in doctors: a systematic review. Occup. Med. (Lond.) 69, 9–21. doi: 10.1093/occmed/kqz004 30753715

[ref27] Kim B. Jee S. Lee J. An S. Lee S. M. (2018). Relationships between social support and student burnout: a meta-analytic approach. Stress. Health 34, 127–134. doi: 10.1002/smi.2771, PMID: 28639354

[ref28] Le T. P. Hsu T. Raposa E. B. (2021). Effects of natural mentoring relationships on college students' mental health: the role of emotion regulation. Am. J. Community Psychol. 68, 167–176. doi: 10.1002/ajcp.12504, PMID: 33823061

[ref29] Leiter M. P. Maslach C. (2009). Nurse turnover: the mediating role of burnout. J. Nurs. Manag. 17, 331–339. doi: 10.1111/j.1365-2834.2009.01004.x, PMID: 19426369

[ref30] Liu S. You J. Ying J. Li X. Shi Q. (2020). Emotion reactivity, nonsuicidal self-injury, and regulatory emotional self-efficacy: a moderated mediation model of suicide ideation. J. Affect. Disord. 266, 82–89. doi: 10.1016/j.jad.2020.01.083 32056950

[ref31] Lopez-Angulo Y. Saez-Delgado F. Mella-Norambuena J. Bernardo A. B. Diaz-Mujica A. (2022). Predictive model of the dropout intention of Chilean university students. Front. Psychol. 13:893894. doi: 10.3389/fpsyg.2022.893894, PMID: 36710762 PMC9881479

[ref32] Mao C. Lin M. Shen S. Li Y. Xie Z. Li P. (2022). Latent profiles of emotion regulation strategies associated with alexithymia, nonsuicidal self-injury and resilience among nursing students. Stress. Health 38, 69–78. doi: 10.1002/smi.3075 34152072

[ref33] Maroco J. Assuncao H. Harju-Luukkainen H. Lin S. W. Sit P. S. Cheung K. C.. (2020). Predictors of academic efficacy and dropout intention in university students: can engagement suppress burnout? PLoS One 15:e0239816. doi: 10.1371/journal.pone.0239816, PMID: 33119598 PMC7595383

[ref34] Mashburn A. J. (2000). A psychological process of college student dropout. J. Coll. Stud. Retent. 2, 173–190. doi: 10.2190/U2QB-52J9-GHGP-6LEE

[ref35] Matthew H. (2018). Processes of natural mentoring that promote underrepresented students***’***educational attainment: a theoretical model. Am. J. Community Psychol. 62, 150–162. doi: 10.1002/ajcp.12251 29873814

[ref36] Morelli M. Chirumbolo A. Baiocco R. Cattelino E. (2023). Self-regulated learning self-efficacy, motivation, and intention to drop-out: the moderating role of friendships at university. Curr. Psychol. 42, 15589–15599. doi: 10.1007/s12144-022-02834-4

[ref37] Muthén L. Muthén B. (2012). Mplus user’s guide (7th ed.). Available online at: https://www.mendeley.com/catalogue/9d069b7e-1057-3c13-925a-26a3a05b8ac7/ (Accessed March 23, 2024).

[ref38] Newman L. A. Madaus J. W. Lalor A. R. Javitz H. S. (2020). Effect of accessing supports on higher education persistence of students with disabilities. J. Divers. High. Educ. 14, 353–363. doi: 10.1037/dhe0000170

[ref39] Noy S. Ray R. (2012). Graduate students***’*** perceptions of their advisors: is there systematic disadvantage in mentorship? J. High. Educ. 83, 876–914. doi: 10.1080/00221546.2012.11777273

[ref40] O’Neill L. D. Wallstedt B. Eika B. Hartvigsen J. (2011). Factors associated with dropout in medical education: a literature review. Med. Educ. 45, 440–454. doi: 10.1111/j.13652923.2010.03898.x 21426375

[ref41] Pedditzi M. L. (2024). School satisfaction and self-efficacy in adolescents and intention to drop out of school. Int. J. Environ. Res. Public Health 21:111. doi: 10.3390/ijerph21010111, PMID: 38248573 PMC10815692

[ref42] Pedditzi M. L. Fadda R. Lucarelli L. (2022). Risk and protective factors associated with student distress and school dropout: a comparison between the perspectives of preadolescents, parents, and teachers. Int. J. Environ. Res. Public Health 19:12589. doi: 10.3390/ijerph191912589, PMID: 36231889 PMC9565153

[ref43] Peng P. Chen S. Hao Y. He L. Wang Q. Zhou Y.. (2023). Network of burnout, depression, anxiety, and dropout intention in medical undergraduates. Int. J. Soc. Psychiatry 69, 1520–1531. doi: 10.1177/00207640231166629 37092762

[ref44] Peng P. Yang W. F. Liu Y. Chen S. Wang Y. Yang Q.. (2022). High prevalence and risk factors of dropout intention among Chinese medical postgraduates. Med. Educ. Online 27:2058866. doi: 10.1080/10872981.2022.2058866, PMID: 35356865 PMC8979499

[ref45] Preacher K. J. Hayes A. F. (2008). Asymptotic and resampling strategies for assessing and comparing indirect effects in multiple mediator models. Behav. Res. Methods 40:879. doi: 10.3758/brm.40.3.879, PMID: 18697684

[ref46] Raposa E. B. Erickson L. D. Hagler M. Rhodes J. E. (2018). How economic disadvantage affects the availability and nature of mentoring relationships during the transition to adulthood. Am. J. Community Psychol. 61, 191–203. doi: 10.1002/ajcp.12228, PMID: 29400907 PMC5837955

[ref47] Rimm-Kaufman S. E. Baroody A. E. Larsen R. A. Curby T. W. Abry T. (2015). To what extent do teacher-student interaction quality and student gender contribute to fifth graders' engagement in mathematics learning? J. Educ. Psychol. 107, 170–185. doi: 10.1037/A0037252

[ref48] Rosales-Ricardo Y. Rizzo-Chunga F. Mocha-Bonilla J. Ferreira J. P. (2021). Prevalence of burnout syndrome in university students: a systematic review. Salud Mental 44, 91–102. doi: 10.17711/sm.0185-3325.2021.013

[ref49] Shah R. Goldstein S. M. (2006). Use of structural equation modeling in operations management research: looking back and forward. J. Oper. Manag. 24, 148–169. doi: 10.1016/j.jom.2005.05.001

[ref50] Stamm M. Buddeberg-Fischer B. (2011). The impact of mentoring during postgraduate training on doctors' career success. Med. Educ. 45, 488–496. doi: 10.1111/j.1365-2923.2010.03857.x 21486324

[ref51] Stinebrickner R. Stinebrickner T. (2014). Academic performance and college dropout: using longitudinal expectations data to estimate a learning model. J. Labor Econ. 32, 601–644. doi: 10.1086/675308

[ref52] Su W. Qi Q. Yuan S. (2022). A moderated mediation model of academic supervisor developmental feedback and postgraduate student creativity: evidence from China. Behav. Sci. 12:484. doi: 10.3390/bs12120484, PMID: 36546967 PMC9774826

[ref53] Su X. Yang Y. Ren Y. Li J. Wang Q. (2023). Mental distress and professional commitment among Chinese medical postgraduate students: a moderated mediation of psychological capital and the supervisor-postgraduate relationship. Psychol. Health Med. 28, 2579–2595. doi: 10.1080/13548506.2023.2224581, PMID: 37332157

[ref54] Sui W. Gong X. Zhuang Y. (2021). The mediating role of regulatory emotional self-efficacy on negative emotions during the COVID-19 pandemic: a cross-sectional study. Int. J. Ment. Health Nurs. 30, 759–771. doi: 10.1111/inm.12830, PMID: 33586868 PMC8013741

[ref55] Tinto V. (1975). Dropout from higher education: a theoretical synthesis of recent research. Rev. Educ. Res. 45, 89–125. doi: 10.3102/00346543045001089

[ref56] Trolian T. L. Parker E. T. (2017). Moderating influences of student-faculty interactions on students*’* graduate and professional school aspirations. J. Coll. Stud. Dev. 58, 1261–1267. doi: 10.1353/csd.2017.0098

[ref57] Vanderweele T. J. (2015). Mediation analysis: a practitioner's guide. Annu. Rev. Public Health 37, 17–32. doi: 10.1146/annurev-publhealth-032315-021402 26653405

[ref58] Wang P. Y. Lin P. H. Lin C. Y. Yang S. Y. Chen K. L. (2020). Does interpersonal interaction really improve emotion, sleep quality, and self-efficacy among junior college students? Int. J. Environ. Res. Public Health 17:4542. doi: 10.3390/ijerph17124542, PMID: 32599755 PMC7345085

[ref59] Wang M. Wang Y. Fang M. Zhang S. Li Y. Cao D.. (2023). Style and influencing factors of tutors-postgraduates*’* interactions in Chinese medical colleges: a cross-sectional survey in Heilongjiang Province. BMC Med. Educ. 23:305. doi: 10.1186/s12909-023-04291-4 37131172 PMC10152608

[ref60] Wang Q. Xin Z. Zhang H. Du J. Wang M. (2022). The effect of the supervisor-student relationship on academic procrastination: the chain-mediating role of academic self-efficacy and learning adaptation. Int. J. Environ. Res. Public Health 19:621. doi: 10.3390/ijerph19052621, PMID: 35270310 PMC8909498

[ref61] West C. Dyrbye L. Satele D. Sloan J. Shanafelt T. (2012). Concurrent validity of single-item measures of emotional exhaustion and depersonalization in burnout assessment. J. Gen. Intern. Med. 27, 1445–1452. doi: 10.1007/s11606-012-2015-7, PMID: 22362127 PMC3475833

[ref62] Willis B. Carmichael K. D. (2011). The lived experience of late-stage doctoral student attrition in counselor education. Qual. Rep. 16, 192–207. doi: 10.46743/2160-3715/2011.1046

[ref63] Xiao Y. Wu X. H. Huang Y. H. Zhu S. Y. (2021). Cultivation of compound ability of postgraduates with medical professional degree: the importance of double tutor system. Postgrad. Med. J. 98:postgradmedj – 2021 – 139779. doi: 10.1136/postgradmedj-2021-139779, PMID: 33837128

[ref64] Yao H. Chen S. Gu X. (2022). The impact of parenting styles on undergraduate students' emotion regulation: the mediating role of academic-social student-faculty interaction. Front. Psychol. 13:972006. doi: 10.3389/fpsyg.2022.972006 36275311 PMC9585973

[ref65] Yu X. Zhao W. Wu X. (2017). Empirical analysis on the status and influence ofthe supervisor-students relationship in universities. J. Tianjin Univ. 19, 157–161.

[ref66] Yuan G. Xu W. Liu Z. Liu C. Li W. An Y. (2018). Dispositional mindfulness, posttraumatic stress disorder symptoms and academic burnout in Chinese adolescents following a tornado: the role of mediation through regulatory emotional self-efficacy. J. Aggress. Maltreat. Trauma 27, 487–504. doi: 10.1080/10926771.2018.1433258

[ref67] Yujie W. Kai D. Yi L. (2013). Revision of the scale of regulatory emotional self-efficacy. J. Guangzhou Univ. 12, 45–50.

[ref68] Zeng Y. Fu Y. Zhang Y. Jiang Y. Liu J. Li J. (2023). Emotion regulation in undergraduate nursing students: a latent profile analysis. Nurse Educ. Pract. 71:103722. doi: 10.1016/j.nepr.2023.103722, PMID: 37467600

[ref69] Zeng B. Zhao J. Zou L. Yang X. Zhang X. Wang W.. (2018). Depressive symptoms, post-traumatic stress symptoms and suicide risk among graduate students: the mediating influence of emotional regulatory self-efficacy. Psychiatry Res. 264, 224–230. doi: 10.1016/j.psychres.2018.03.022, PMID: 29655115

[ref70] Zhang X. Yue H. Sun J. Liu M. Li C. Bao H. (2022). Regulatory emotional self-efficacy and psychological distress among medical students: multiple mediating roles of interpersonal adaptation and self-acceptance. BMC Med. Educ. 22:283. doi: 10.1186/s12909-022-03338-2, PMID: 35421953 PMC9011952

[ref71] Zhang X. Zhou Q. Vivor N. K. Liu W. Cao J. Wang S. (2023). Sequential mediation of early temperament and eating behaviors in the pathways from feeding practices to childhood overweight and obesity. Front. Public Health 11:1122645. doi: 10.3389/fpubh.2023.1122645, PMID: 37766743 PMC10520502

